# Relationship between job stress, thinking style and symptoms of post-traumatic stress disorder in mental health nurses

**DOI:** 10.3389/fpubh.2022.979138

**Published:** 2022-09-15

**Authors:** Wuyi Liu, Lin Sun, Xunbao Yin, Huan Zhao, Guohui Zhu, Bo Lian, Hongwei Sun

**Affiliations:** ^1^School of Public Health, Weifang Medical University, Weifang, China; ^2^School of Psychology, Weifang Medical University, Weifang, China; ^3^School of Teacher Education, Weifang University, Weifang, China; ^4^Depression Treatment Center, Weifang Mental Health Center, Weifang, China; ^5^School of Biological Sciences and Technology, Weifang Medical University, Weifang, China

**Keywords:** job stress, thinking style, post-traumatic stress disorder, mental health nurses, moderation model

## Abstract

**Introduction:**

Mental health nurses are often exposed to stressful events which may lead to feeling of stress in their daily work, and this feeling has a profound impact on nurses' mental health.

**Aim:**

This study aimed to evaluate the relationship between job stress, thinking style and symptoms of post-traumatic stress disorder (PTSD) of mental health nurses, and to explore the mechanism of job stress and thinking style on symptoms of PTSD.

**Method:**

This cross-sectional study collected related data of 351 mental health nurses in China, and the data was analyzed by PROCESS macro for SPSS.

**Results:**

The results showed that 18.2% of mental health nurses had the symptoms of PTSD. Thinking style (monarchic thinking style, anarchic thinking style and external thinking style) played a moderating role in the predictive effects of job stress on the symptoms of PTSD.

**Discussion:**

The research pointed out the relationship between job stress and symptom of PTSD, and clarified the critical role of thinking style among mental health nurses.

**Implications for practice:**

It is recommended that organizations should enact effective policy and intervention programs to reduce job stress and PTSD symptoms of mental health nurses which may improve their mental health level.

## Introduction

Post-traumatic stress disorder (PTSD) is an anxiety disorder characterized by the re-experiencing of an extremely traumatic event accompanied by symptoms of increased arousal and by avoidance of stimuli associated with the trauma (1). PTSD is a worldwide problem. Studies have showed that the prevalence rate of PTSD is between 0 and 17% among mental health care providers (2, 3).

Due to the unpredictable changes in the daily work and the unrealistic demands of patients and their families, a hospital is a stressful workplace for the medical staff (4). Nurses are at risk of PTSD due to indirect and/or direct exposure to traumatic environment when providing care for patients (5). Because of the special working environment, mental health nurses will not only get first-hand information of patients' extreme trauma experience and face more pressure of daily work, but also experience more trauma events than nurses of other specialties (such as medical and surgical); and they are more vulnerable to violence and attack (6), and even have a higher probability of witnessing or experiencing suicide of others (7). A review of the literature shows that the attack rate of workers in the mental health field ranges from 14 to 64% every 6 months, and frequent exposure to workplace violence and threats of violence increase the stress level of staff and the risk of PTSD (3). Mental health nurses are faced with various job stress, with the risk of suffering from PTSD (8). PTSD will lead to an increase in turnover rate, and reduce the productivity and service quality of medical services. When nurses experience PTSD, the attrition rate, absenteeism rate and patient care quality of nurses will generally decline (9). Therefore, we should attach great importance to PTSD of mental health nurses. However, we found that not all mental health nurses have PTSD symptoms, which indicates that there are other factors can moderate their stress and trauma.

One possible factor leading to PTSD is work-related stress. Hilton et al. (10) showed that workplace violence is an important stressor for nurses. A review found that discordant working environment, such as tense relationship with colleagues and lack of organizational support, will increase the severity of PTSD of nurses (5). The above factors such as workplace violence, traumatic events and disharmonious working environment can be included in the overall job stress of nurses. Usually, high levels of job stress will have a negative impact on nurses' mental health, and even lead to PTSD (11). It has been found that interpersonal conflict in job stress can significantly predict PTSD symptom severity, and Lee et al. (12) put forward a tentative model in his research on mental health nurses, indicating high level of job stress will lead to increased PTSD.

Intellectual style is an umbrella term of style constructs such as learning styles, cognitive styles and thinking styles, which refers to the preferred way for a person to deal with information and tasks (13). Sternberg's (14) theory of mental self-government is one of the most recent theories of intellectual style. This theory believes that the individuals are similar to the government, and they will choose their preferred ways to use their abilities, that is, thinking styles. Therefore, Sternberg imitated the setting of government agencies and identified thinking styles into 13 different types according to five dimensions: functions, forms, levels, scopes and leanings. Subsequently, Zhang and Sternberg (13) divided the 13 thinking styles into three types as follows: the first type of thinking style including legislative, judicial, global, hierarchical, and liberal styles; the second type of thinking style including executive, local, monarchic, and conservative styles; and the third type of thinking style including oligarchic, anarchic, internal, and external styles.

Briefly, legislative style individuals like to create their own rules, do things in their favorite ways, and have the most creativity. Individuals with judicial style are more like commentators, who like to evaluate rules, procedures and other things, and also like to evaluate and judge the performance of others. Global style people like to start from the whole, pay more attention to the whole picture of an issue, and like to deal with abstract issues, but tend to ignore details. A person with hierarchical style likes to deal with multiple affairs at the same time, and sort the tasks to be dealt with prioritized according to his valuing. Individuals with liberal style like to go beyond existing rules and procedures, like the uncertain working environment, and have a high tolerance for change. Executive style people's thinking mode is the opposite of legislative style people's, they like to follow rules and implement tasks with established guidelines. Individuals with local style like to deal with concrete and detailed work, but it is easy to lose sight of global changes. People who exhibit a predominantly monarchic style tend to focus on one job at a time, and they are relatively unself-aware, intolerant and inflexible. Individuals with a predominantly conservative style like to existing rules and procedures, like familiar life and work, and don't like to change the status quo. People with equal competitive style like to deal with multiple objectives at the same time without setting priorities, and they are usually indecisive. Anarchic style people like to work at will, and they are too flexible and do not have firm set of rules upon which to base priorities. Internal style people are task-oriented and like to work alone. They are introverted, unsociable and dislike dealing with people's relationships. External style people are more extroverted than internal style people. They are more sensitive to interpersonal relationships and like to do things with others or finish work in groups.

There is a certain conceptual connection between thinking style and PTSD, and both of them have a strong cognitive component. Early theorists thought that the development of PTSD was caused by the contradiction between the individual's preexisting schema (a cognitive structure) and experience of traumatic events. Usually, the people with extreme views, either positive or negative, are more vulnerable to PTSD (15, 16). We can understand that when a person finds that traumatic events are completely contradictory to his previous schema or expectation, this huge difference may cause him to doubt and produce PTSD. On the other hand, when a person thinks that he is entirely incompetent and the world is completely dangerous, his previous schema or belief will be strengthened by traumatic events, thus increasing fear and producing PTSD. Therefore, to a certain extent, the degree of PTSD of individuals depends on their cognition. Thinking styles are cognitive because they represent people's preferred ways of processing tasks and using abilities. At present, it is found that there is a certain relationship between thinking style and individual mental health. After controlling age and sex, seven of the 13 thinking styles (judicial, local, hierarchical, monarchic, anarchic, internal, and external) had significant associations with mental health (17). In nursing undergraduates, hierarchical and anarchic thinking styles can predict mental health (18). Internal thinking style can predict depression of college students (19). However, the contributions of thinking styles to PTSD are yet to be examined.

Taken together, stress is thought to contribute to mental illness or psychosomatic dysfunction (20), but the theoretical model of stress holds that this relationship must be moderated by some factors. Moreover, individuals with different thinking styles will choose different ways to deal with the same task (stress) and bring different results. And hence, we hypothesized that job stress is associated to PTSD, and thinking style plays a significant role in moderating the relationship between them. In the present study, in order to explore the potential factors involved in PTSD, so as reduce PTSD symptoms of mental health nurses and improve their mental health level, we investigated the relationship between job stress, thinking style and PTSD of 351 mental health nurses, as well as the mechanism of job stress and thinking style on symptoms of PTSD based a cross-sectional study. Our findings will provide a theoretical basis for institutions to develop effective policies and intervention programs to reduce work stress and PTSD symptoms of mental health nurses.

## Methods

### Research design

This descriptive, cross-sectional study was conducted in 3 mental health hospitals in Shandong province, China. Data was collected by the questionnaires over a 4-month period in 2021. Participants were given enough time (usually within 20 min) to complete the questionnaire and asked to remain anonymous.

### Participants

The study comprised 3 mental health hospitals from Shandong Province, China. Sample inclusion criteria included those who had been working for more than 1 year, volunteered to participate in the survey, and provided direct patient care. Sample exclusion criteria included nurses from other places for further study and nurses in management roles. The questionnaires were distributed to 400 nurses, 49 of which were excluded because the answer options were the same or missing. Finally, a total of 351 nurses were enrolled in this study (effective response rate of 87.75%).

### Instruments

A self-report, structured questionnaire was designed for the current study. The questionnaire consists of four parts.

#### Demographic data

Participants' sociodemographic characteristics such as age, highest nursing degree, years of nursing experience, job position, and training in coping with stress were collected.

#### Nurse job stressors inventory

Job stress was measured by a questionnaire developed by Li and Liu (21) on the nurses' experience of job stress. The inventory includes five subscales and comprises 35 items, where the subscales consist of items related to the following sources of stress: (1) professional and career issues, (2) workload and time pressure, (3) resource and environmental problems, (4) patient care and interaction and (8) interpersonal relationships and management issues.

This inventory is a 4-point Likert response scale, where a score of one indicates “no stress” and a score of four indicates “great stress”. The higher score indicates the higher level of job stress. This measure is the most widely used instrument for measuring nursing job stress in China and also has good reliability and validity. Cronbach's alpha values for the original questionnaire were 0.98, and 0.94 in the current study, demonstrating good internal consistency reliability.

#### Thinking style inventory-revised II (TSI-R2)

Thinking style was measured by a questionnaire developed by Sternberg et al. (22) on the individual's thinking style. The TSI-R2 consisting of 65 items was used to assess the 13 thinking styles delineated by Sternberg (14), and it can measure 13 thinking styles: legislative, executive, judicial, hierarchical, oligarchic, monarchic, anarchic, global, local, internal, external, liberal, and conservative styles. The TSI-R2 is a 7-point Likert response scale, with 1 denoting the item does not reflect the way they normally carry out their tasks at all and seven indicating that it does so extremely well. The TSI-R2 has been validated in Chinese context (23, 24) and demonstrated acceptable reliability and validity. In the present study, Cronbach's alpha value was 0.91, and the coefficients of the 13 styles ranged between 0.65 and 0.76, demonstrating good internal consistency reliability.

#### PTSD checklist-civilian version (PCL-C)

PTSD was measured with the Post-traumatic Checklist Civilian Version (PCL-C) (25), which is an easily administered self-report rating scale for assessing DSM-IV-TR symptoms of PTSD. The instrument is divided into three parts: the first part consists of items relating to intrusive thoughts and feelings, the second part consists of those relating to avoidance and the last part relates to hyper-arousal. The scale is composed of 17 items and has a Likert score system ranging from 1 to 5. The total score ranges from 17 to 85, and the higher the total score, the more serious the degree of PTSD. In this study, the total score <44 is negative, and 44–85 is PTSD positive (12, 26). The Chinese translation of PCL-C has high reliability and validity, and has been widely used to access the symptoms and severity of PTSD among different populations including Chinese (27, 28). In this study, the PCL had an internal consistency of 0.91 for all 17 items, demonstrating good internal consistency reliability.

### Data analyses

All analyses were conducted using SPSS version 22.0 (SPSS Chicago, IBM, USA). Descriptive statistics were used to analyze the participants' characteristics and basic statistical results. The chi-square test was conducted to examine differences in the different PTSD groups. The Pearson correlation test was used to examine relationships between variables. The hypothesized moderated mediation model was analyzed through the PROCESS macro for SPSS (29), based on ordinary least squares regression. In this study, Mode 1 in Hayes's PROCESS was used to test the moderating effect. The bias-corrected 95% confidence interval (CI) was calculated with 5,000 bootstrapping resamples. The effect was statistically significant at *p* = 0.05 if the interval did not cover zero.

## Results

### Descriptive statistics

All survey participants were female (*n* = 351), 126 were over age 35 (35.90%). College (*n* = 128, 36.50%) and undergraduate (*n* = 170, 48.40%) education accounted for 84.9% of the overall sample. Most survey participants' Job position were nurses (*n* = 291, 82.90%), and approximately one-third of the participants had 3–4 years of nursing experience (*n* = 101, 28.80%). Most participants (*n* = 218, 62.10%) had not participated in stress relief training or activities. According to the scoring rule, those with a score of 44 or above were defined having positive PTSD symptoms. Of the sample, 64 participants were screened to have PTSD symptoms in this study, and the proportion of PTSD was 18.20%. The chi-square test showed that job position and training in coping with stress were related to PTSD (*p* < 0.01; Table 1).

**Table 1:** Sociodemographic characteristics and PTSD symptoms among the overall sample.

	**PTSD-**		**PTSD**+			
	***n***	**%**	***n***	**%**	***χ^2^***	***p***
Age					2.332	0.675
19 or younger	4	1.1	0	0		
20–24	31	8.8	5	1.4		
25–29	73	20.8	20	5.7		
30–34	74	21.1	18	5.1		
35 or older	105	29.9	21	6.0		
Highest nursing degree					5.928	0.205
Technical secondary school education	27	7.7	11	3.1		
High school diploma	6	1.7	1	0.3		
College degree	109	31.1	19	5.4		
Scholar degree	137	39.0	33	9.4		
Master degree or above	8	2.3	0	0		
Years of nursing experience					2.872	0.579
1–2	77	21.9	12	3.4		
3–4	78	22.2	23	6.6		
5–6	49	14.0	10	2.8		
7–8	24	6.8	6	1.7		
9 or above	59	16.8	13	3.7		
Job position					8.174*	0.043
Nursing worker	22	6.3	5	1.4		
Nurse	232	66.1	59	16.8		
Deputy head nurse	11	3.1	0	0		
Head nurse	22	6.3	0	0		
Training in coping with stress					6.949**	0.008
Yes	118	33.6	15	4.3		
No	169	48.1	49	14.0		

^*^p < 0.05,

^**^p < 0.01.

### Correlations between the study variables

Pearson correlation analysis showed there were correlations among variables (Table 2). Five positive significant correlations were found between job stress and global thinking style (*r* = 0.242, *p* < 0.001), job stress and local thinking style (*r* = 0.244, *p* < 0.001), job stress and PTSD (*r* = 0.425, *p* < 0.001), PTSD and judicial thinking style (*r* = 0.116, *p* < 0.05), and PTSD and oligarchic thinking style (*r* = 0.135, *p* < 0.05). Five negative significant correlations were found between job stress and external thinking style (*r* = −0.127, *p* < 0.05), PTSD and legislative thinking style (*r* = −0.108, *p* < 0.05), PTSD and executive thinking style (*r* = −0.124, *p* < 0.05), PTSD and hierarchical thinking style (*r* = −0.122, *p* < 0.05), as well as PTSD and external thinking style (*r* = −0.286, *p* < 0.001).

**Table 2:** Correlation between job stress, thinking style and PTSD.

**Variable**		**1**	**15**
1 job stress		—	0.425***
	2 legislative	−0.035	−0.108*
	3 executive	0.067	−0.124*
	4 judicial	0.005	0.116*
	5 monarchic	−0.061	0.015
	6 hierarchical	0.014	−0.122*
	7 oligarchic	0.079	0.135*
Thinking style	8 anarchic	0.089	0.035
	9 global	0.242***	0.024
	10 local	0.244***	0.011
	11 internal	−0.068	−0.024
	12 external	−0.127*	−0.286***
	13 liberal	−0.040	−0.036
	14 conservative	0.019	−0.096
15 PTSD		0.425***	—

^*^p < 0.05,

^***^p < 0.001.

### Job stress and PTSD: Moderated moderation analysis

We hypothesized that thinking style might play a moderator role in the relation between job stress and PTSD. According to Hayes's (29) SPSS macro program PROCESS, the parameters of the equation was estimated with Model 1 (Table 3). In this model, job stress was the independent variable (X), 13 kinds of thinking styles were individually as a moderator (M), and PTSD was the dependent variable (Y). Age, highest nursing degree, years of nursing experience, job position, and training in coping with stress were controlled, and study variables were standardized in the model.

**Table 3:** Moderated Effect of Job stress on PTSD.

		**β**	***t***	***p***	**95%CI**	***R^2^***	***F***
Equation 1						0.278	16.454***
Job stress		0.489	9.595***	0.000	[0.389, 0.589]		
Monarchic thinking style		0.134	2.625**	0.009	[0.034, 0.234]		
Job stress × Monarchic thinking style		0.144	2.910**	0.004	[0.047, 0.242]		
	Age	−0.063	−0.805	0.422	[−0.217, 0.091]		
	Highest nursing degree	−0.114	−2.342*	0.020	[−0.210, −0.018]		
	Years of nursing experience	−0.004	−0.051	0.959	[−0.164, 0.156]		
	Job position	−0.201	−3.810***	0.000	[−0.305, −0.097]		
	Training in coping with stress	0.040	0.839	0.402	[−0.054, 0.134]		
Equation 2						0.263	15.265***
Job stress		0.499	9.734***	0.000	[0.398, 0.599]		
Anarchic thinking style		−0.009	−0.188	0.851	[−0.101, 0.084]		
Job stress × Anarchic thinking style		0.109	2.143*	0.033	[0.009, 0.209]		
	Age	−0.067	−0.858	0.392	[−0.221, 0.087]		
	Highest nursing degree	−0.135	−2.766**	0.006	[−0.230, −0.039]		
	Years of nursing experience	−0.001	−0.016	0.987	[−0.162, 0.160]		
	Job position	−0.163	−3.108**	0.002	[−0.267, −0.060]		
	Training in coping with stress	0.023	0.485	0.628	[−0.071, 0.117]		
Equation 3						0.294	17.785***
Job stress		0.468	9.198***	0.000	[0.368, 0.568]		
External thinking style		−0.185	−3.826***	0.000	[−0.280, −0.090]		
Job stress × External thinking style		−0.106	−2.453*	0.015	[−0.191, −0.020]		
	Age	−0.034	−0.447	0.656	[−0.184, 0.116]		
	Highest nursing degree	−0.118	−2.350*	0.019	[−0.216, −0.019]		
	Years of nursing experience	−0.014	−0.170	0.865	[−0.171, 0.144]		
	Job position	−0.159	−3.092**	0.002	[−0.260, −0.058]		
	Training in coping with stress	0.021	0.443	0.658	[−0.071, 0.113]		

^*^p < 0.05,

^**^p < 0.01,

^***^p < 0.001.

Equation 1 examined the moderating effects of monarchic thinking style (M); Equation 2 examined the moderating effects of anarchic thinking style (M); and equation 3 examined the moderating effects of external thinking style (M). Results of regression analyses showed that the prediction of job stress on PTSD, the interaction of job stress and monarchic thinking style, the interaction of job stress and anarchic thinking style, and the interaction of job stress and external thinking style were all significant. Monarchic thinking style exerted a moderating effect on the relationship between job stress and PTSD (β = 0.144, *t* = 2.910, 95%CI [0.047, 0.242], *P* = 0.004). Anarchic thinking style exerted a moderating effect on the relationship between job stress and PTSD (β = 0.109, *t* = 2.143, 95%CI [0.009, 0.209], *P* = 0.033). External thinking style exerted a moderating effect on the relationship between job stress and PTSD (β = −0.106, *t* = −2.453, 95%CI [−0.191, −0.020], *P* = 0.015). According to Hayes (30), the moderated mediating model was valid.

**Figure 1:**
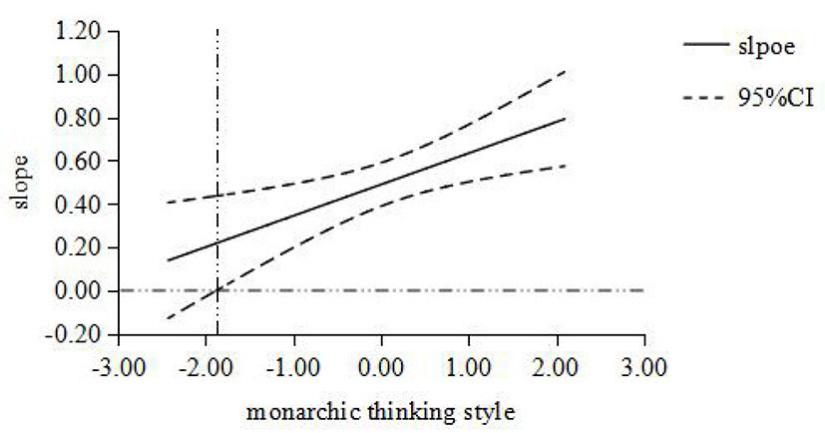
The moderating role of monarchic thinking style between job stress and PTSD. The abscissa is the moderating variable of monarchic thinking style, and the ordinate represents the change of regression coefficient (i.e. slope) in regression equation with PTSD as dependent variable, job stress as independent variable and monarchic thinking style as moderating variable. All variables are standardized; The middle line is the point estimate, and the upper and lower lines are the values of 95% confidence interval.

**Figure 2:**
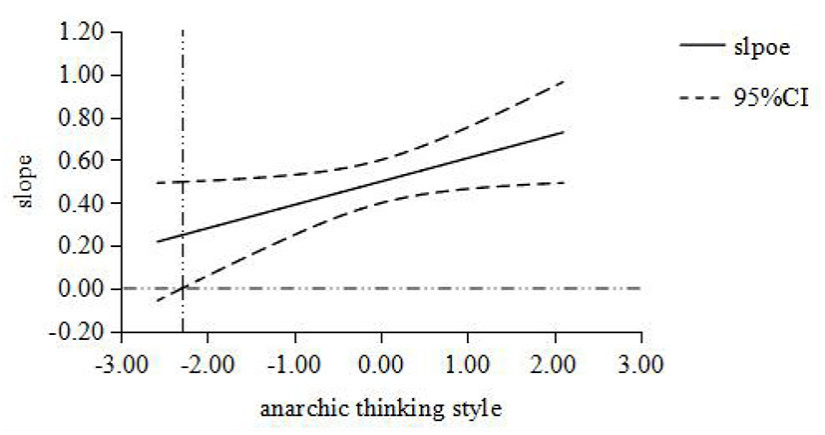
The moderating role of anarchic thinking style between job stress and PTSD. The abscissa is the moderating variable of anarchic thinking style, and the ordinate represents the change of regression coefficient (i.e. slope) in regression equation with PTSD as dependent variable, job stress as independent variable and anarchic thinking style as moderating variable. All variables are standardized; The middle line is the point estimate, and the upper and lower lines are the values of 95% confidence interval.

**Figure 3:**
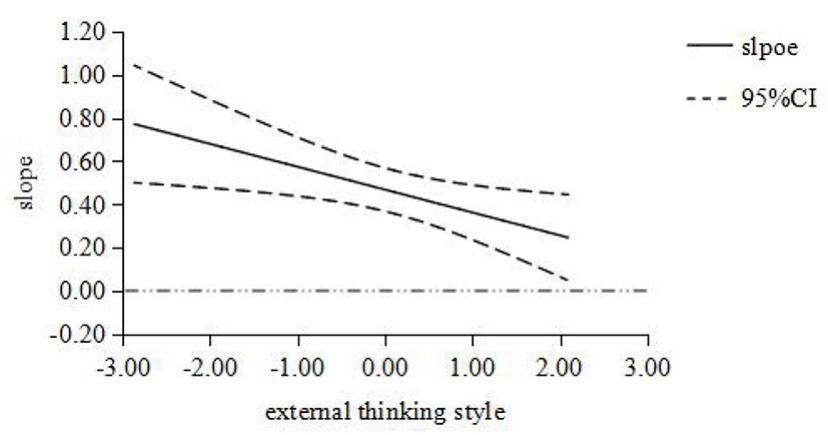
The moderating role of external thinking style between job stress and PTSD. The abscissa is the moderating variable of external thinking style, and the ordinate represents the change of regression coefficient (i.e., slope) in regression equation with PTSD as dependent variable, job stress as independent variable and external thinking style as moderating variable. All variables are standardized; The middle line is the point estimate, and the upper and lower lines are the values of 95% confidence interval.

Furthermore, the Johnson-Neyman simple slope test (31) was used to analyze the moderator role of thinking style. When the moderator variable monarchic thinking style was set at [-1.879, 2.096], the simple slope was [0.218, 0.791], which is obviously not contain 0; When the moderator variable anarchic thinking style was set at [-2.297, 2.106], the simple slope was [0.249, 0.728], which is obviously not contain 0; When the moderator variable external thinking style was set at [-2.866, 2.096], the simple slope was [0.247, 0.771], which is obviously not contain 0. Then the moderating effect diagram of thinking style between job stress and PTSD was made (Figures 1–3). The results of simple slope test showed that the greater the job stress, the more serious the PTSD degree of the subjects; moreover, the influence of job stress on PTSD will be weakened, when the subject with low monarchic thinking style, low anarchic thinking style and high external thinking style.

## Discussion

In our study, 18.20% of mental health nurses were screened out PTSD symptoms, which was similar to previous reports. For example, the detection rate of PTSD in mental health hospital staff in Botswana (south central Africa) was 18.4% (2, 3), 16% among Canadian psychiatrists measured by PCL-5 (10), and 14–17% among Australian mental health nurses measured by severity scoring method and symptom endorsement scoring method (12). In a word, PTSD is a serious issue affecting nurses all over the world that should be taken into consideration.

In the present study, we observed a significant correlation between job stress and PTSD, which was consistent with our initial hypothesis and key for verifying the moderating effect of thinking style. This result was also consistent with most previous studies, which have reported a positive correlation between job stress and PTSD (10, 12, 32). Mental health nurses are often exposed to violence toward, critical events (e.g., physical threats, assaults, or deaths) and chronic stressors (e.g., patients drink from the toilet or smear feces) in their daily work. Some chronic stressors are tied to violent events (e.g., staff injury incurred while removing patients from rooms) (10, 33). Therefore, it will bring pressure to mental health nurses and increase PTSD symptoms. In addition, mental health nurses are not only experiencing the consumer/carer stressors, but also bearing the stress of work teams and organizations, such as staff conflict and bullying, high workloads and lack of organizational support (34, 35). Generally, adequate social support is identified as a protective factor to against PTSD (36), whereas negative social support is a risk factor (37). Individuals who have access to social support in stressful situations, seem to be better off (38, 39). Therefore, mental health nurses may not get more support from colleagues and organizations, which may increase their job stress and PTSD symptoms. However, this hypothesis needs to be verified in future studies. In addition, the negative perceptions of patient care, such as the negative patient outcomes and mistakes in treating patients, cause stress and distress for nurses, affecting their mental health and PTSD symptoms (5, 40). Work overload and stress of nurses in psychiatric department will contribute to burnout development (41), thus aggravate PTSD symptoms.

Consistent with our assumptions, thinking style moderated the relationship between job stress and PTSD. This finding shows that individuals with low monarchic thinking style, low anarchic thinking style and high external thinking style can alleviate the relationship between work stress and PTSD, and reduce the risk of PTSD. Lavoie et al. (42) believe that extraversion is negatively associated with PTSD symptoms, and being energetic or positive (extraversion) will be a protective factor. Individuals who use external thinking style prefer to cooperate with others to deal with problems, which helps them reduce anxiety, job stress and PTSD. People with anarchic thinking style are too flexible, and they may be hostile to existing rules and interventions from others (24). This may make them more vulnerable to stress at work, which may aggravate PTSD symptoms. Therefore, people with low-level anarchic thinking style will feel less job stress and hardly showing PTSD symptoms. People with monarchic thinking style like to focus on one thing in a certain period of time, but it is difficult for nurses to focus on one job because of their busy work. This kind of work with opposite thinking style may make them feel great stress and affect their mental health and PTSD symptoms. Therefore, people with low-level monarchic thinking style can adapt to nurses' work and reduce job stress and PTSD symptoms.

With the novel coronavirus pandemic (COVID-19) in China, health care workers, including nursing workers, usually need to stay on the front line of fighting the epidemic. These extreme working conditions and the high number of cases and deaths have resulted in a heavy burden on medical staff (43). In addition, existing studies have shown that such outbreaks can lead to the development of PTSD (44–46). Data from previous pandemics suggest that PTSD rates increase among health care workers during health care crises, especially those who work directly with infected patients (47). Studies in the United States confirmed earlier findings, with approximately 23% of healthcare workers reporting probable PTSD and 57% scoring above the cutoff value on PTSD screening measures (48, 49). Nurses, who work with COVID-19 patients, showed more severe symptoms of traumatic stress (50). Notably, participants who had a relative, friend, or colleague who died from COVID-19 were more likely to present with high levels of PTSD (46). In contrast, there was no difference in personal infection history among those without PTSD, suggesting highlights that exposure to high-risk work environments, such as direct care of infected patients, is not a major determinant of adverse psychological outcomes. With the development of COVID-19, its fatality rate becomes lower and lower. Hence, we hypothesize that the incidence of high levels of PTSD in nurses due to death from COVID-19 would gradually decrease or even disappear. In the present study, we did not consider the influence of COVID-19 factors mainly because the current mortality rate of COVID-19 is very low, and the stress of COVID-19 prevention work can be attributed to job stress.

To sum up, in the prevention and intervention of PTSD for mental health nurses, we can start from two aspects, one is to relieve job stress, and the other is to enhance the cultivation of thinking style. Reducing the work intensity, violent exposure and the frequency of traumatic events of mental health nurses, promoting harmonious working environment and interpersonal relationship, and strengthening organizational and social support are all helpful to relieve job stress. According to Sternberg's idea, the individual's thinking style is not static. We can carry out targeted training for mental health nurses, train them to use external thinking style more in their work, and reduce the use of monarchic and anarchic thinking style. In addition, mindfulness-based stress reduction training (51) and rumination training (52) can be added to the training, which will help to relieve stress and PTSD symptoms.

### Limitations

The current study possesses some limitations of note. First, all data came from patients' self-reports. Though self-reports can reflect an individual's true feelings, but our results may have also been influenced by recall bias. Second, most of the mental health nurses in these three hospitals were women, and the results of our investigation were all women. As a result, our sample cannot analyze the gender differences of mental health nurses. Finally, this was a cross-sectional and retrospective study, making it impossible to determine causal relationship between variables. Furthermore, PTSD was measured without criterion A. And hence, in the future research, experiments can be conducted to test the causality, and diagnostic criteria can be further used to diagnose PTSD.

## Conclusion

Our findings demonstrate that there is a significant relationship between job stress and PTSD among mental health nurses, while thinking style (monarchic, anarchic and external) can play a moderating role between them. That is, when nurses have low monarchic thinking style, low anarchic thinking style and high external thinking style, the influence of job stress on PTSD will be weakened. Analysis the factors related to mental health of mental health nurses could lead to more effective prevention for adverse health outcomes and better use of resources to promote positive outcomes. More psychological support should be provided for mental health nurses.

## Relevance for clinical practice

The job stress regularly faced by mental health nurses in the course of their work can, if unaddressed, affect their mental health. As nurses are crucial to providing quality care for the general population, identifying and instituting effective prevention and intervention strategies to improve psychological outcomes for nurses is essential. First of all, we can provide a relatively safe and friendly working environment for mental health nurses, and reduce the probability of PTSD from the root cause. Secondly, we can evaluate the thinking style of mental health nurses, and carry out regular education and training, so as to encourage them to use the thinking style that is more conducive to handling job. Experiencing stress events does not only bring negative consequences, but it is our ultimate goal to help nurses gain post-traumatic growth.

## Data availability statement

The datasets presented in this article are not readily available to be shared at this time because the data also form part of an ongoing study. Requests to access the datasets should be directed to Wuyi Liu, liuwuyi01@163.com.

## Ethics statement

The studies involving human participants were reviewed and approved by Ethics Committee of Weifang Medical College. The patients/participants provided their written informed consent to participate in this study.

## Author contributions

WL: formal analysis, writing original draft, and review and editing. LS: conception and design and methodology. XY: investigation and review and editing. HZ and GZ: investigation. BL: methodology. HS: conceptualization, investigation, and supervision. All authors contributed to the article and approved the submitted version.

## Funding

This work was supported by the Medical Education Research Project of Chinese Medical Association (Grant Number 20A1209), the Education Teaching Reform and Research Fund of Weifang Medical University (Grant Number 2019YB023), the National Natural Science Foundation of China (NSFC) (Grant Number 82101588), the Surface Project of Natural Science Foundation of Shandong Province (Grant Number ZR2020MC218), the Institute of Psychology, Chinese Academy of Sciences (Grant Number GJ202002), the Open Fund of the Center of China- US Sports Economics and Health Engineering of Shandong (Grant Number SDCA20191011), the Science and Technology Development Program of Traditional Chinese Medicine in Shandong Province (Grant Number 2019WS594/202002010572), and the Education and Teaching Reform Project of the Psychology and Education Reference Committee of the Ministry of Education (Grant Number 20221013).

## Conflict of interest

The authors declare that the research was conducted in the absence of any commercial or financial relationships that could be construed as a potential conflict of interest.

## Publisher's note

All claims expressed in this article are solely those of the authors and do not necessarily represent those of their affiliated organizations, or those of the publisher, the editors and the reviewers. Any product that may be evaluated in this article, or claim that may be made by its manufacturer, is not guaranteed or endorsed by the publisher.
